# The Occurrence of Pain-Induced Depression Is Different between Rat Models of Inflammatory and Neuropathic Pain

**DOI:** 10.3390/jcm10174016

**Published:** 2021-09-06

**Authors:** Yung-Chi Hsu, Kuo-Hsing Ma, Shu-Lin Guo, Bo-Feng Lin, Chien-Sung Tsai, Chun-Chang Yeh

**Affiliations:** 1 Department of Anesthesiology, Tri-Service General Hospital, National Defense Medical Center, Taipei 114, Taiwan; x0939778570@gmail.com (Y.-C.H.); pjdadsl@gmail.com (S.-L.G.); bflin0114@gmail.com (B.-F.L.); 2 Integrated Pain Management Center, Tri-Service General Hospital, National Defense Medical Center, Taipei 114, Taiwan; 3 Department of Biology and Anatomy, National Defense Medical Center, Taipei 114, Taiwan; kuohsing91@yahoo.com.tw; 4 Department of Anesthesiology, Cathay General Hospital, Taipei 106, Taiwan; 5 School of Medicine, Fu Jen Catholic University, New Taipei City 242, Taiwan; 6 Division of Cardiovascular Surgery, Department of Surgery, Tri-Service General Hospital, National Defense Medical Center, Taipei 114, Taiwan; sung1500@mail.ndmctsgh.edu.tw; 7 Department and Graduate Institute of Pharmacology, National Defense Medical Center, Taipei 114, Taiwan

**Keywords:** depression-like behavior, inflammatory pain, neuropathic pain, positron emission tomography, serotonin transporter

## Abstract

Various pain conditions may be associated with depressed mood. However, the effect of inflammatory or neuropathic pain on depression-like behavior and its associated time frame has not been well established in rat models. This frontward study investigated the differences in pain behavior, depression-like behavior, and serotonin transporter (SERT) distribution in the brain between rats subjected to spared nerve injury (SNI)-induced neuropathic pain or complete Freund’s adjuvant (CFA)-induced inflammatory pain. A dynamic plantar aesthesiometer and an acetone spray test were used to evaluate mechanical and cold allodynia responses, and depression-like behavior was examined using a forced swimming test and sucrose preference test. We also investigated SERT expression by using positron emission tomography. We found that the inflammation-induced pain was less severe than neuropathic pain from days 3 to 28 after induced pain; however, the CFA-injected rats exhibited more noticeable depression-like behavior and had significantly reduced SERT expression in the brain regions (thalamus and striatum) at an early stage (on days 14, 21, and 28 in two groups of CFA-injected rats versus day 28 in SNI rats). We speculated that not only the pain response after initial injury but also the subsequent neuroinflammation may have been the crucial factors influencing depression-like behavior in rats.

## 1. Introduction

Pain is one of the main reasons that patients seek medical treatment, and it is a major clinical, social, and economic problem. Chronic pain can cause sleep disturbance, anxiety, and depression [1]. Neuropathic pain is affected by peripheral and central sensory pathways, which might partly explain the high comorbidity rates of neuropathic pain and psychological conditions such as depression [2]. Neuropathic pain can take the form of hyperalgesia, allodynia, or persistent spontaneous pain after a nerve injury [3]. Inflammatory pain is a natural physiological response to tissue injury or infection. The chemical mediators responsible for tissue inflammation act on nociceptive nerve endings and can lead to the development of allodynia, hyperalgesia, or depression [4]. Currently, neuropathic pain seems to be associated with the complexity of neuropathic symptoms, poor outcomes, and tough treatment challenges. In addition, clinical neuropathic pain is usually accompanied by anxiety and depression [5]. In addition, anti-inflammatory medication is the main treatment option for inflammatory pain, but such medication often offers little relief from such pain and can also cause side effects.

Current clinical guidelines for treating neuropathic and inflammatory pain are somewhat problematic due to the existence of various complicated contributing factors [6]. Recent studies have shown that the absorption of serotonin is related to some neuropsychiatric diseases and disorders, such as Parkinson’s disease, depression, and substance use disorder [7]. Studies have shown that the serotonin transporter (SERT) and changes in serotonin are related to chronic central neuropathic pain [8]. Researchers have used positron emission tomography (PET) images of the SERT in the brains of rats in investigations of neuropsychiatric disorders and indicated that the SERT is involved in regulating the aforementioned neuropsychiatric diseases and disorders [9]. However, to our knowledge, no studies have compared models of neuropathic pain and inflammatory pain by using PET images of the SERT in the brains of rats. 

The effects of neuropathic pain and inflammatory pain on depression development is a topic worth exploring; neuropathic pain and inflammatory pain have been investigated in many studies, but few have compared them. In this study, the relationships between pain behavior, depression-like behavior, and the SERT in rat brains were explored using two rat pain models.

## 2. Materials and Methods

### 2.1. Animals

Our experimental protocol was approved by the Animal Care and Use Committee (IACUC-19-112) of the National Defense Medical Center (Taipei, Taiwan, Republic of China), and the experiments were conducted in accordance with the Guide for the Care and Use of Laboratory Animals by the US National Institutes of Health. Seven-week-old male Wistar rats (200–250 g, BioLASCO, Taipei, Taiwan) were used in this study. The rats were housed individually, maintained in a 12 h light–dark cycle (lights on: 07:00; lights off: 19:00), and given ad libitum access to food and water for 7 days so that they could adjust to their new surroundings prior to the experiments. The rats were randomly assigned to one of four experimental groups (6 in each group): the sham group, spared nerve injury (SNI) group, complete Freund’s adjuvant (CFA) group, and diluted CFA group. A dynamic plantar aesthesiometer (DPA) and an acetone spray test were used to evaluate responses to mechanical and cold allodynia, respectively. Paw thickness was measured using a digital vernier caliper, and depression-like behavior was investigated using the forced swimming test (FST) and sucrose preference test (SPT). We also investigated brain SERT expression in the four rat groups by using animal PET.

### 2.2. SNI Model of Neuropathic Pain

The protocol for the SNI group has been described in detail elsewhere [10]. SNI was induced through the ligation of the common peroneal and tibial branches of the left sciatic nerve under 1.5% isoflurane anesthesia, but the sural nerve was not ligated. The same protocol was applied in the sham and CFA groups but without nerve ligation.

### 2.3. CFA Model of Inflammatory Pain

Inflammatory pain was induced in rats through injection with CFA (F5881; 1 mg/mL of heat-killed and dried Mycobacterium tuberculosis; Sigma-Aldrich) using the method described by McCarson [11]. The rats in the two CFA groups were anesthetised using 1.5% isoflurane. The footpad plantar surface was cleaned with 70% ethanol, and a needle was inserted into the footpad to deliver the 100-μL CFA solution (0.5 mg/mL for the CFA group and 0.25 mg/mL for the diluted CFA group). The operator’s thumb was placed lightly on the footpad during needle withdrawal, and pressure was maintained for at least 10 s. The sham and SNI groups underwent the same procedure but were injected with normal saline.

### 2.4. Mechanical Allodynia Test

Mechanical allodynia was measured using a DPA (Ugo Basile, Comerio, Italy) over a period of 28 days [12]. Each rat was placed in a plastic cage for 15 min of adaptation time. Subsequently, the filament was placed below the territory of the sural nerve at the palmar surface of the left ipsilateral hind paw, and incremental pressure (maximum 50 g, 2.5 g per s) was applied until the rat withdrew its hind paw. The pressure recorded when the rat withdrew its hind paw was determined to be the paw withdrawal threshold (PWT). The results are expressed as the mean of three independent measurements.

### 2.5. Cold Allodynia Test

An acetone spray was used to assess cold allodynia over a period of 28 days. The rats were placed in transparent chambers and acclimated for 15 min. A drop of acetone (100 μL) was gently applied to the palmar surface of the ipsilateral hind paw. The latency time—that is, the time the animal spent lifting, licking, or shaking its paw over 2 min—was recorded. The results are expressed as the mean of three independent measurements [13].

### 2.6. Hind Paw Dorsoventral Thickness

Over a period of 28 days, a digital vernier caliper was used to measure paw thickness. The following equation was used: paw thickness (as a percentage of the contralateral side) = paw thickness (surgical side)/paw thickness (contralateral side).

### 2.7. Measurement of TIMP-1 and CXCL1 Levels in Footpad Tissues

The footpad tissues were homogenized in a lysis buffer and centrifuged for 25 min at 18,000× *g* (4 °C). The protein concentration was quantitated by a BCA Protein Assay Kit (Thermo scientific, Rockford, IL, USA). We used aliquots of footpad homogenates to detect tissue chemokine levels. The TIMP-1 and CXCL1 levels in the footpad tissues were measured using ELISA kits from R&D Systems (Abingdon, UK) according to the manufacturer’s protocol [14].

### 2.8. FST Procedures

Depression-like behavior was examined using the FST [15]. Rats were forced to swim in a vertical plastic cylinder (diameter: 30 cm; height: 50 cm) containing 30 cm of water maintained at 23 °C. In a 5-min duration, the score for the predominant behavior in each 5-s period was recorded for every rat. Each rat was dried with a towel, and the water was changed after each test.

### 2.9. SPT Procedures

Depression-like behavior was also examined using the SPT [16]. For such tests, rats were deprived of water and food for 24 h. After deprivation, all rats were given free access to the liquid in two bottles, one containing water and one containing 1% sucrose solution. After 1 h, the volumes of water and sucrose solution consumed by the rats were recorded. The following equation was used: sucrose preference index (%) = sucrose consumption volume/(sucrose consumption volume  +  water consumption volume) × 100.

### 2.10. PET Imaging of the SERT

Radioligand 4-[18F]-ADAM, provided by Tri-Service General Hospital, was synthesized using the approach described by Peng et al. [17].

Specifically, 4-[18F]-ADAM (14.8–18.5 MBq; 0.5–0.6 mCi) was injected through the tail vein of rats for PET imaging. PET image acquisition, which followed the procedure described in a previous study [18], was conducted with the rats under isoflurane-inhalation–induced anesthesia. One hour after radioligand injection, PET imaging was performed using a PET scanner (BIOPET 105, BIOSCAN) (Concorde MicroSystem, Knoxville, TN, USA). The energy window was set to 250–700 keV. The Fourier rebinning algorithm and two-dimensional filtered back-projection (ramp filter with cutoff at Nyquist frequency) were applied to reconstruct the images. All images were analyzed using AMIDE software. The volumes of interest for the midbrain, hypothalamus, caudate putamen, hippocampus and frontal cortex thalamus, striatum, and cerebellum were defined on reconstructed and summated PET images according to an MRI rat brain atlas to determine the rats’ anatomical boundaries. The specific uptake ratio (SUR) was derived as (target region − cerebellum)/cerebellum [18].

### 2.11. Statistical Analysis

SPSS (version 22.0; SPSS Inc., Chicago, IL, USA) was used for the statistical tests, and the results are expressed as mean ± standard error. Comparisons between means were conducted using repeated-measures analysis of variance. Differences between groups were analyzed using a post hoc test or Tukey’s honestly significant difference test. The results were considered statistically significant if *p* was <0.05.

## 3. Results

### 3.1. Mechanical and Cold Allodynia Tests after SNI and CFA Injection

**Figure 1:**
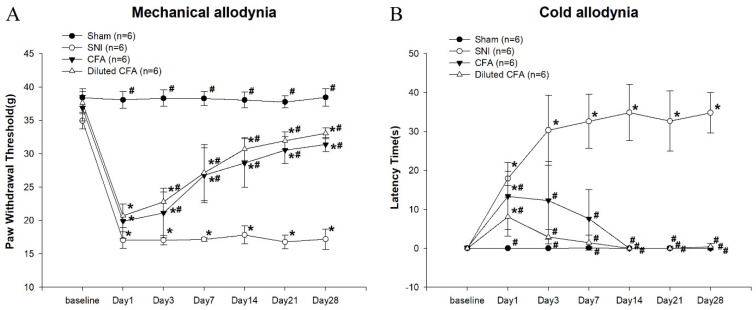
(**A**) PWT for the mechanical allodynia test. Rats were randomly assigned to one of four experimental groups (*n* = 6): the sham (without nerve injury, injection of normal saline) group (●), SNI (nerve injury, injection of normal saline) group (○), CFA (without nerve injury, injection of CFA) group (▼), and diluted CFA (without nerve injury, injection of diluted CFA) group (△). Mechanical allodynia responses were examined using a DPA. * *p*  <  0.05 compared with the sham group. # *p*  <  0.05 compared with the SNI group. (F(3, 20) = 5.443; F(3,20) = 169.517; F(3,20) =116.519; F(3,20) = 50.419; F(3,20) = 87.124; F(3,20) = 246.681; F(3,20) = 374.424). (**B**) Latency time of the cold allodynia test. Behavioral responses in the four groups. * *p*  <  0.05 compared with the sham group. # *p*  <  0.05 compared with the SNI group. (F(3,20) = 2.458; F(3,20) = 13.777; F(3,20) = 24.516; F(3,20) = 50.872; F(3,20) = 140.446; F(3,20) =106.188; F(3,20) =263.093).

Mechanical allodynia responses were examined (Figure 1A), and comparisons between the SNI, CFA, and diluted CFA groups were performed. From day 1 to day 28, the PWTs of the SNI, CFA, and diluted CFA groups were significantly reduced after pain was induced. The SNI group maintained a low PWT with persistent pain throughout the 28 day observation period. In contrast to that of the SNI group, the PWT of the CFA and diluted CFA groups recovered by approximately 80% over time. PWTs were not significantly different between the CFA and diluted CFA groups throughout the 28 day period. 

Regarding cold allodynia tests (Figure 1B), in the SNI group, the latency time was considerably long throughout the experiment. However, the CFA and diluted CFA groups responded only to acetone stimulation in the first week.

### 3.2. Hind Paw Thickness (Dorsoventral)

**Figure 2:**
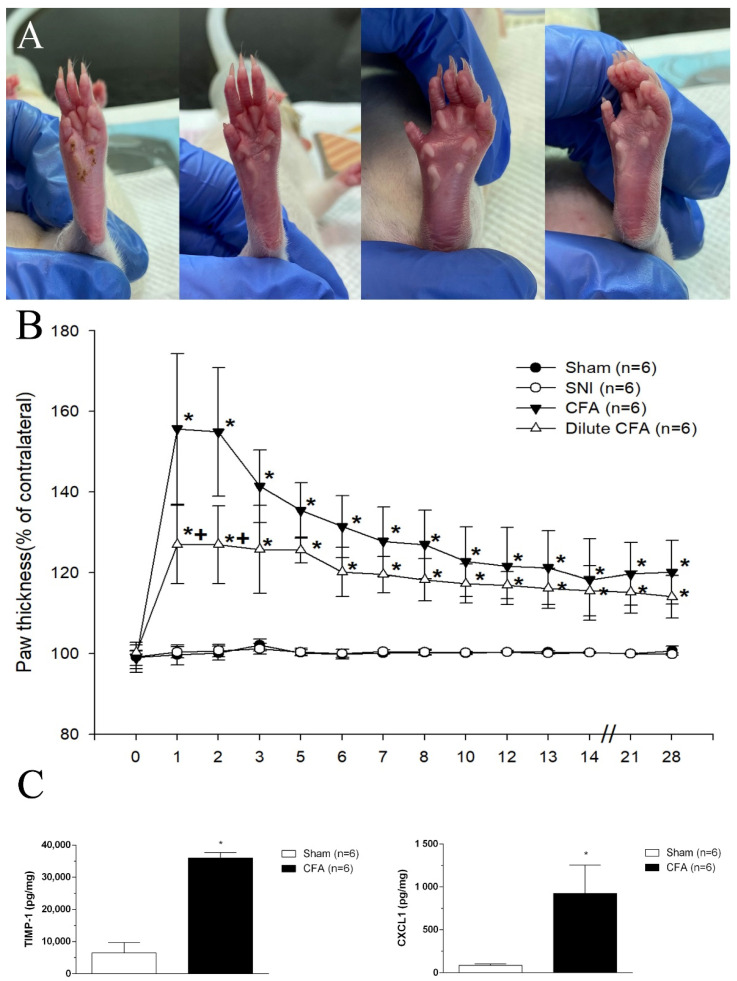
(**A**) Photographs of the footpads of rats 1 day after the injection of 100 μL of saline or CFA. From left to right are the sham, SNI, CFA, and diluted CFA groups. (**B**) Paw thickness changes (%) = (surgical side/contralateral side). * *p*  <  0.05 compared with the sham group. + *p*  <  0.05 compared with the SNI group, *n* = 6. (**C**) The enhanced chemokine expression of TMP-1 and CXCL1 induced by CFA on day 1. * *p*  <  0.05 compared with the sham group. (F(1,10) = 6.493).

After injection, in the CFA groups, the left hind paw thickness increased significantly, and such paws remained swollen from day 1 to day 28; such increases were not observed in the SNI group. Photographs of the footpads of rats 1 day after injection are presented in Figure 2A. Figure 2B presents changes in paw thickness (%) from baseline to day 28.

### 3.3. CFA Induced the Increases of TIMP-1 and CXCL1 Levels in Footpad Tissues

Immune cells are infiltrated to inflammatory tissues by chemokines. TIMP-1 and CXCL1 are the crucial chemokines regulated by NF-κB or STAT1 [19]. Indeed, we found that significant increases in TIMP-1 and CXCL1 levels in the footpad tissues were induced by CFA (Figure 2C).

### 3.4. Depression-like Behavior

**Figure 3:**
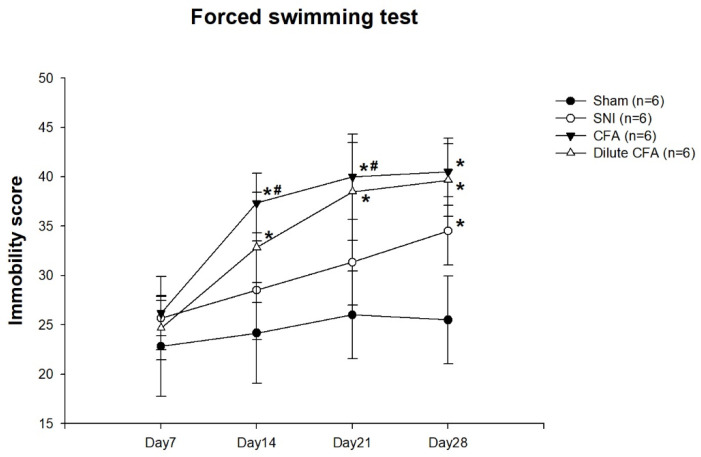
FST rat immobility scores on days 7, 14, 21, and 28. Results are expressed as mean ± standard deviation. * *p*  <  0.05 compared with the sham group. # *p*  <  0.05 compared with the SNI group. Each group had six rats. (F(3,20) = 0.976; F(3,20) = 8.419; F(3,20) = 12.46; F(3,20) = 20.085).

FST immobility scores are shown in Figure 3. On day 7, no obvious difference between the four groups was observed. On days 14 and 21, the immobility scores of the CFA and diluted CFA group were markedly higher than those of the sham and SNI groups. On day 28, the immobility score of the SNI group was increased, almost to the level of the CFA and diluted CFA groups.

**Figure 4:**
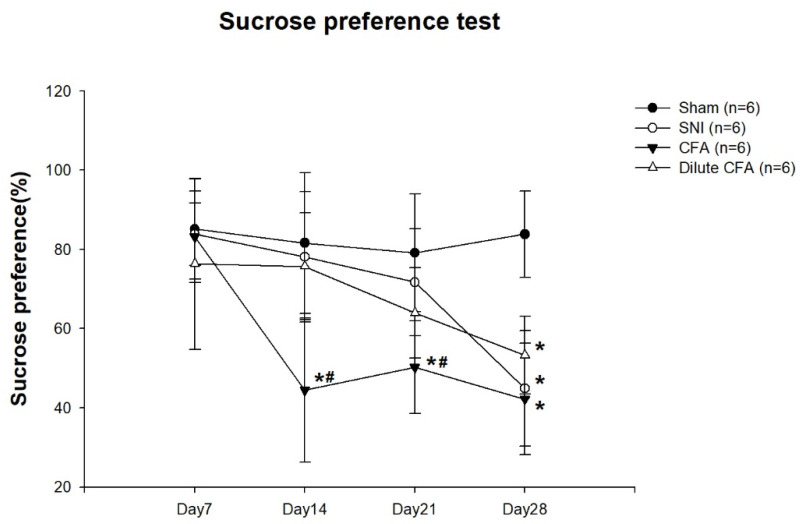
SPT percentage on days 7, 14, 21, and 28. Results are expressed as mean ± standard deviation. * *p*  <  0.05 compared with the sham group. # *p*  <  0.05 compared with the SNI group. Each group had six rats. (F(3,20) = 0.547; F(3,20) = 6.428; F(3,20) = 5.46; F(3,20) = 14.023).

Sucrose preferences are presented as percentages in Figure 4. On day 7, no obvious differences between the four groups were observed. On days 14 and 21, the sucrose preference of the CFA group was markedly lower than those of the other groups. On day 28, the sucrose preferences of the three experimental groups were markedly lower than that of the sham group.

### 3.5. PET Images of SERT Expression in the Brains of Rats

**Figure 5:**
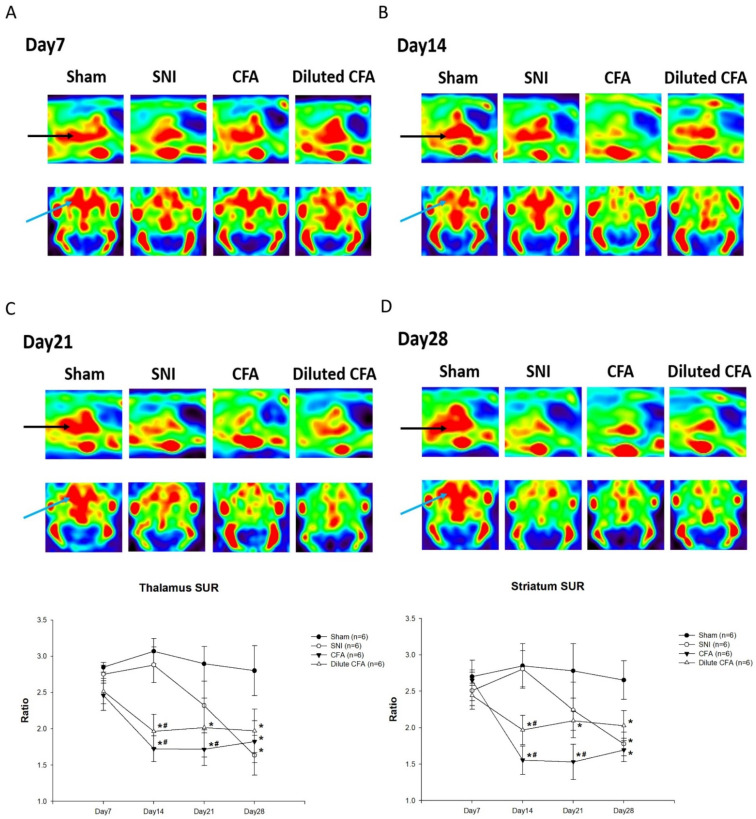
PET images of rat brains and SURs on (**A**) day 7, (**B**) day 14, (**C**) day 21, and (**D**) day 28. The black arrow indicates the thalamus region, and the blue arrow indicates the striatum region. Results are expressed as mean ± standard deviation. * *p*  <  0.05 compared with the sham group. # *p*  <  0.05 compared with the SNI group. Each group had six rats. Thalamus SUR (F(3,20) = 11.354; F(3,20) = 59.193; F(3,20) = 15.721; F(3,20) = 17.149), striatum SUR (F(3,20) = 2.522; F(3,20) = 41.563; F(3,20) = 17.488; F(3,20) =27.399).

SERT distributions in the rat brains are displayed in Figure 5. On day 7 (Figure 5A), no obvious differences between the four groups were observed. On day 14 (Figure 5B), no significant difference in SERT distribution was noted between the sham and SNI groups, but marked changes were observed in the CFA and diluted CFA groups in terms of SERT expression in the striatum and thalamus. A similar phenomenon was noted on day 21 (Figure 5C). On day 28 (Figure 5D), the SERT distribution in the SNI group was reduced from baseline and close to the level of the CFA and diluted CFA groups. The SURs of the SERT in the thalamus and striatum of the rats in the four groups are presented in Figure 5.

## 4. Discussion

This study revealed that rats subjected to neuropathic pain after SNI or inflammatory pain after CFA injection exhibited pain behavior. Compared with baseline measurements, a significant reduction in PWT and a longer latency time was observed in two groups. The mechanical and cold allodynia responses of the rats in the SNI group were stronger and more persistent (28 days) than those of the rats in the CFA groups. The PWT of the CFA groups increased on day 3 and returned to a level that was 80% of the sham group level 28 days after CFA injection. Moreover, the time the CFA group rats spend lifting, licking, or shaking their paws was the same as that of the sham group 14 days after CFA injection. After treatment, the rats in both the SNI and the CFA groups had decreased sucrose consumption and increased immobility time in the FST. The depression-like behaviors of the rats were observed earlier in the CFA group (day 14) than in the SNI group (day 28). Additionally, the left hind paw thickness increased significantly from day 1 to day 28 in the CFA group after injection, but such an increase was not apparent in the SNI group. From a clinical perspective, pain severity and chronicity may predict the earlier onset of depression. However, this was not observed in these experiments. On the basis of our results, we assume that the extent of inflammation, rather than pain intensity, could be the key factor influencing depression-like behavior in rats.

The mechanical and cold allodynia responses of the SNI group are consistent with those in another study [20]. Furthermore, the behaviors we observed after CFA-induced inflammatory pain are in line with those noted by Leiguarda [21]. Szabo-Pardi et al. and Cook et al. have hypothesized that SNI and CFA pain have similar pathways and mechanisms [22,23]. They assumed that macrophages in the dorsal root ganglion and microglia in the spinal cord are activated early in response to nerve injury or peripheral inflammation and remain active throughout the condition’s pathogenesis [22,23]. The chronic activation of macrophages and microglia facilitates the recruitment of other immune cells, leading to long-term neuroimmune interactions and neuroinflammation [22,23]. This reciprocal signaling has long-term effects on neural plasticity [22]. Q wu et al. also think that inflammatory pain and neuropathic pain share common mechanisms [24].

Sheng et al. surmised that pain and depression occur simultaneously due to maladaptive changes in neural plasticity. These changes occur in overlapping brain regions, including the striatum, thalamus, posterior cingulate cortex, and anterior cingulate cortex, and the relevant brain pathways involved in pain and depression are located in these regions [25]. A study revealed that brain regions involved in modulating pain perception are best identified using fMRI and PET [26]. Growing evidence on neural maladaptive plasticity changes indicates that the sensation of neuropathic pain is transmitted through the peripheral and central sensory pathways [27].

Our results related to the depression-like behavior of rats subjected to SNI- or CFA-induced pain agree with those in relevant studies [28,29]. Zhang et al. reported that FST immobility time increased on days 7 and 14 after CFA injection in rats. Furthermore, the SPT percentage decreased on day 14 after CFA injection [28]. Their results are similar to ours. Fang et al. also reported that immobility time significantly increased and SPT percentage decreased on day 42 in rats subjected to SNI [29]. In those rats, depression-like behaviors after SNI were observed later than they were in the rats subjected to SNI in our experiment. Overall, depressive-like behaviors manifested earlier in rats given CFA than in those subjected to SNI. 

Inflammation affects the brain and behavior; in one study, its specific effects were apparent in the neurocircuits and neurotransmitter systems that appear to be most affected by inflammation in patients with depression or other psychiatric disorders [30]. Subpopulations of patients with depression have higher levels of proinflammatory cytokines, acute phase proteins, chemokines, and cellular adhesion molecules. Peripherally released inflammatory cytokines can access the brain and influence all the pathophysiological domains relevant to depression [31]. Furthermore, brain–immune system interactions have been confirmed in previous studies, and they can provide an explanation as to how cytokines affect the brain. Capuron L et al. speculated that there are three pathways involved in immune system to brain signaling. In the humoral pathway, inflammatory cytokines pass through the leaking area of the blood–brain barrier. In the neural pathway, the nucleus of the tractus solitarius is evoked by sensory afferent stimulation. In the cellular pathway, monocytes cross the blood–brain barrier due to their attraction to monocyte chemoattractant protein-1, which is produced by microglia [32]. However, the immunology of inflammation in depression needs to be further explored. 

Our findings on the left hind paw thickness of rats given CFA agree with those in a previous study [21]. The left hind paw thickness increased significantly from day 1 to day 28 after CFA injection, but such an increase was not observed in rats subjected to SNI. The phenomena of constant and progressive hind paw edema and increased dermal thickness are indicative of a persistent inflammatory condition.

Previous studies have elucidated the mechanisms by which the inflammation or pain interacts with depression in different animal models. Blockading of inflammatory pathway components or anti-inflammatory drugs have been shown to reduce depressive symptoms in rats in a CFA-induced inflammatory pain model [28,33], supporting the crucial role of inflammation in the CFA model. In addition, AM Le et al. showed that an anti-nociceptive compound can alleviate pain hypersensitivity and depression-like behavior in the SNI-induced neuropathic pain model [34]. In this study, we further found that decreased SUR of the SERT expression in the thalamus and striatum was consistent with the appearance of depression-like behavior in the CFA-induced pain model, which manifested earlier than in the SNI-induced pain model, implying that inflammation rather than pain is responsible for the depressive behavior.

Two theories could explain why the depression-like behaviors of rats were observed earlier in the CFA model than in the SNI model. First, according to the concept of a brain–immune system interaction pathway, depression induced by SNI was mainly associated with neural and cellular pathways, not humoral pathways [32]. In addition to neural and cellular pathways, in the CFA model, the pathway of brain–immune system interactions contributed numerous inflammatory cytokines via humoral pathways. Second, the persistent swelling of hind paws in both groups of CFA injection rats throughout the 28 days’ observation represented non-resolving chronic inflammation that impacted the brain-immune system continuously. Therefore, we assumed that the effect of neural and cellular brain–immune system interactions induced by CFA was more potent than the effects induced by SNI. This explains why the depression-like behaviors of the rats were observed earlier in the CFA model than in the SNI model. 

The SERT is a major regulator of serotonin homeostasis. Early alterations in serotonergic signaling have been linked to diverse neurodevelopmental disorders, including depression [35]. Selvaraj, et al. reported that depressed patients had lowered SERT in some brain regions including the thalamus and striatum regions, which are similar to our findings in rats [36]. The timing of the significant decrease in the SUR of the SERT in the thalamus and striatum was compatible with the manifestation of depression-like behavior in our experimental groups. Furthermore, as we previously stated, certain brain regions are related to pain and depression [25,26]. We assume that the decrease in the SUR of the SERT in the thalamus and striatum was associated with depression-like behaviors in our experimental groups. 

This study has some limitations. We investigated the differences in pain behavior, depression-like behavior, and serotonin transporter (SERT) distribution in the brain between rats subjected to SNI-induced neuropathic pain or CFA-induced inflammatory pain. Since the performance of cytokines plays an important role in pain and behavior, it is necessary to analyze the difference in cytokines in animals under the two animal models in this study. However, considering that excessive stimulation and pressure manipulation may affect the analysis of behavior end-point, and under standards of IACUC for research ethics (5R: replace, reduce, refine, reuse, and rehabilitate), we were not allowed to sacrifice more animals to obtain experimental data, so we did not analyze the performance of cytokines in this study. The analysis of behaviors at the end point of this research is still the central issue, so we believe that CFA-induced inflammatory pain inducing depression-like behavior more quickly than SNI-induced pain is believable in rats.

## 5. Conclusions

We found that rats in the CFA-induced inflammatory pain group had a lower pain response to non-nociceptive stimulus than rats in SNI-induced neuropathic pain, but were more depressed at an early stage as well as having a significant reduction of SERT expression in certain brain regions. According to our experimental results, not only initial pain but also the subsequent inflammatory cascades may have been the crucial factor influencing the depression-like behavior of rats.

Little is understood about the management of chronic-pain-induced depression, and a gap remains between fundamental research and the implementation of related findings in clinical settings. Future investigations are necessary to validate our findings and efforts must be devoted to determining the connection between pain and depression. Our findings may provide valuable information for clinicians using anti-inflammation strategies to treat depression caused by chronic pain through the suppression of macrophage and microglial cell activity. Therefore, further research should focus on these connections to identify related therapeutic targets against inflammation-induced pain and associated depression. The various endogenous inflammatory cytokines and exogenous cytokines linked to depression are associated with specific risk factors, and further research in the future may lead to the development of preventive interventions.

## Data Availability

The data presented in this study are available on request from the corresponding author.
